# Histological Characteristics, Fatty Acid Composition of Lipid Fractions, and Cholesterol Content of *Semimembranosus* and *Triceps Brachii* Muscles in Maremmana and Limousine Bovine Breeds

**DOI:** 10.3389/fvets.2017.00089

**Published:** 2017-06-14

**Authors:** Andrea Serra, Giuseppe Conte, Elisabetta Giannessi, Laura Casarosa, Carla Lenzi, Alessandro Baglini, Francesca Ciucci, Alice Cappucci, Marcello Mele

**Affiliations:** ^1^Department of Agriculture, Food and Environment, University of Pisa, Pisa, Italy; ^2^Research Center of Nutraceuticals and Food for Health, University of Pisa, Pisa, Italy; ^3^Department of Veterinary Sciences, University of Pisa, Pisa, Italy

**Keywords:** Maremmana breed, muscle fiber types, histological cell muscle characteristics, fatty acid composition, cholesterol, phospholipids

## Abstract

This study examined the histological properties of *Semimembranosus* and *Triceps brachii* muscle in two different bovine breeds, Maremmana (Ma) (an autochthonous breed from Tuscany, Italy) and Limousine (Lm). The animals were grazed in two adjoining pastures, received the same feed supplementation, and were weighed monthly. The experimental period lasted from weaning (6 months old) to slaughter (19 months old). Muscle samples were collected immediately after slaughter, before carcass cooling. Regarding the histological properties, the number of muscle fibers (TNF), mean sarcolemma perimeter (MSP), cross-sectional area, and total sarcolemma perimeter (TSP) were determined. Samples were also analyzed for proximate composition, fatty acid profile of total lipids (TLs), phospholipids (PLs), and neutral lipids (NLs), and for total cholesterol content. Breed was a significant variation factor for the performance parameter and histological muscle fiber properties. Interestingly, despite that Ma being a less extensively genetically improved breed than Lm, it showed higher weight at slaughter (+18%) and daily weight gain (+19%). Ma also showed smaller muscle fibers than Lm and, consequently, the TSP was higher. This difference affected the lipid fraction distribution (Lm was higher in PLs and lower in NLs than Ma) and, consequently, the fatty acid composition of TLs (Lm was high in polyunsaturated fatty acids, while Ma was high in monounsaturated fatty acids). The results of this experiment highlight the importance of environmental and management conditions on the full expression of genotypic potential.

## Introduction

Bovine breeds are characterized by a specific phenotypic conformation, which is largely due to the effects of extensive genetic improvement (GI) over the last 50 years. With respect to bovine meat breeds, the goal of GI is to improve animal performance to increase the average daily gain and dressing yield. GI is obtained by a quantitative approach based on phenotypic animal measurements and more recently, by molecular selection. Some bovine breeds have been intensively genetically improved, with the result that their conformation perfectly meets the ideal productive characteristics, with a very high rearing and slaughtering performance. Conversely, some autochthonous bovine breeds, due to their original low productivity, have not been genetically improved, and their current characteristics and phenotypic conformation are quite similar to the original ones. Genetic improvements have affected not only the animal productivity but also the meat quality. In fact, by selecting for the phenotypic globosity of animals, a particular kind of muscle fiber has been selected, and the improved breeds show bigger fibers than native breeds (1, 2). The muscle fiber types dramatically affect both the physical and nutritional quality of meat (1, 3). Muscle fibers can be histologically and physiologically classified into red-oxidative and white-glycolytic fibers, and, with respect to contraction velocity, into fast twitch and slow twitch (4, 5). The red-oxidative muscle fibers are smaller than white-glycolytic fibers. In addition, red fibers are resistant to fatigue because the energy for muscle contraction comes from oxidative metabolism, thus a lot of energy (which, conversely, is slowly available) is produced. Thus, red muscle fibers show a great number of mitochondria, myoglobin, and heme iron (2).

The different types of muscle fibers also show a different lipid composition. In fact, especially lipids contained in the sarcolemma, such as phospholipids (PLs) and cholesterol, putatively have a higher content in the smaller fibers (the red ones) due to their longer total sarcolemma perimeter (TSP) (4). PLs play a functional role in the cell, thus they show a characteristic fatty acid composition, high in polyunsaturated fatty acids (PUFA) and low in saturated fatty acids (SFA) compared with triacylglycerols, which represent the main energy storage in the animal organism.

Clearly, the phenotypic conformation, animal performance, and meat quality are also determined by the environment. In fact, genetic types react differently to the rearing system, depending on how they adapt to grazing or to particular soil and climatic conditions. Thus, the rearing system and animal movement might affect the muscle fiber distribution both from a histological and physiological point of view (2).

The aim of this experiment was to study the effect of breed [Maremmana (Ma) and Limousine (Lm)] and type of muscles [*Semimembranosus* (SM) and *Triceps brachii* (TB)] on histological characteristics and lipid composition. Lm is a well-known beef cattle breed, extensively reared in the world and genetically improved. Ma is an autochthonous beef cattle breed reared in Maremma, which is a rural area in the southern Tuscany (Italy). Maremma is characterized by a very low annual precipitation (about 600 mm per year), and high medium and maximum temperatures. These conditions classify Maremma as a marginal area in which the extensive livestock farming is strongly affected by the poor quality and quantity of pasture. To best of our knowledge this is the first experiment to study the muscle of the Ma breed compared with a cosmopolitan breed in terms of the histological characteristics and meat quality.

## Materials and Methods

### Animals and Diets

The study was carried out on 10 female bovines (5 Ma and 5 Lm) from an organic farm located in Grosseto, in southern Tuscany (Italy). To avoid feed competition, the two animal groups were grazed in two adjoining pastures throughout the experimental period. Pasture was sampled once a month throughout experimental period. The chemical and the botanical composition of the pasture are shown in Table 1. When the grass growth was poor [autumn (AU)] or absent (winter), the daily ration of both groups was integrated with hay and concentrate (see Table 1 for the chemical composition). The feeding regimen adopted met the Council Regulation (EC No 834/2007) organic rules for animal rearing and was set to obtain an average daily gain of 1.1 kg. The experiment lasted approximately 1 year, from weaning (6 months old and 210 ± 10 kg in weight) to slaughter. The animals were simultaneously weaned (March 2015); this was aimed at limiting the effect of botanical and chemical composition variability of the pasture throughout the year (Table 1).

**Table 1:** Chemical composition of pasture and feed (g/100 g of DM) and botanical composition of pasture.

	Pasture					Hay	Concentrate
	SP	SU	AU	SE	*P*		
DM (g/100 g of feed)	18.8^b^	33.2^a^	31.8^a^	2.55	<0.01	86.6	89.5
Ether extract	3.6	3.0	2.7	0.27	0.26	1.8	2.1
Crude protein	11.5^a^	7.7^b^	5.1^c^	0.34	<0.01	8.0	14.6
NDF	55.9^c^	62.4^b^	77.1^a^	1.69	<0.01	60.0	15.2
ADF	31.0^c^	39.3^b^	53.2^a^	1.49	<0.01	37.7	6.3
ADL	3.7^c^	7.5^b^	15.0^a^	0.63	<0.01	5.4	0.8
Ashes	5.8	6.1	5.1	0.27	0.14	9.4	6.9
NSC	4.4	6.9	3.2	0.25	0.21	20.8	61.2
FUs/kg of DM	0.7^a^	0.6^b^	0.3^c^	0.01	0.02	0.5	1.0

**Pasture composition (g/100 g DM)**							
Leguminous	21.4^a^	6.0^b^	4.7^b^	3.12	0.02	–	–
Grasses	45.6^b^	76.4^a^	49.8^ab^	7.28	0.03	–	–
Other	33.0^b^	17.6^c^	45.5^a^	7.66	0.04	–	–
Yeld (ton of DM/ha)	1.9^a^	3.9^a^	3.2^a^	2.55	<0.01	–	–

*SP, spring; SU, summer; AU autumn; DM, dry matter; NDF, neutral detergent fiber; ADF, acid detergent fiber; ADL, acid detergent lignin; NSC, no-structural carbohydrates; FUs, fodder units*.

*Grasses were composed by Bromus mollis, Hordeum vulgare, Lolium perenne, Hordeum murinum, Zizania spp., and Poa trivialis; Leguminous were composed by Lotus corniculatus, Vicia faba, Onobrychis viciifolia, and Trifolium pratense; other families were composed by Plantago lanceolata, Convolvulus cneorum, Senecio vulgaris, Picris spp., Potentilla reptans, and Coriandrum sativum*.

*^a,b,c^Means values within a row with different superscript letters are significantly different (*P* ≤ 0.05)*.

Since muscle fiber differentiation and growth are affected by animal age (5), all animals were 19 months of age at the time of slaughter (March–April 2016). Live weights were recorded again just before the slaughter. This study was carried out in accordance with the EU guidelines (EU directive 2010/63) for the care and use of animals in research (Italian official bulletin n. 61, 2014). According to the above reported guidelines (art. 2, letter d), protocol approval by the Ethical Committee of the National Ministry of Health was not necessary, because the experiment was carried out according to the usual livestock husbandry techniques.

The abattoir was a public facility and was less than 10 km from the farm. The animals were slaughtered shortly after arrival.

### Muscle Sampling

The muscle sampling was performed when the animals were slaughtered and before the right-hand side of the carcass had cooled. Whole TB and SM muscles were collected. For the histological determinations, three 1 cm × 1 cm portions with the same length muscle were immediately sampled from each muscle. Each portion was then divided into three parts (proximal, intermediate, and distal) and immediately placed into a 50 mL test tube containing about 40 mL of formalin to preserve the sample until analysis. The sampling for chemical analyses was performed by the same procedure, except that the samples were immediately frozen until analysis.

### Histological Analysis

Samples were fixed in 4% paraformaldehyde in 0.1 M phosphate-buffered saline (pH 7.4), dehydrated, and embedded in paraffin. Sections of 5 µm were cut by a microtome (Reichert-Jung). Sections were stained with Mallory’s Trichrome (Bio-Optica) modified to facilitate microscopic examination. The specimens were assessed under a Leitz Diaplan microscope (Nikon Eclipse Ni) connected to a digital image acquisition system (Nikon digital-sight DS-U1) with the help of the “NIS” program (NIS-Elements BR-4.13.00, Nikon Corporation, Tokyo Japan). For each of the acquired images, the number of muscle fibers (TNF), mean sarcolemma perimeter (MSP), TSP, and cross-sectional area (CSA) were reported.

### Chemical Analysis

The moisture content was determined by weight difference by placing the samples into a ventilated oven at 105°C for 24 h. Crude fat in animal feed was determined by extracting 0.5 g of the sample with petroleum ether using an XT10 Ankom apparatus (Macedon, NY, USA) without acid hydrolysis, according to the AOCS official procedure AM 5-04. Protein content was determined by Kjeldahl methods according to the AOAC official procedure AOAC 981.10. Crude fiber and fiber fraction (neutral detergent fiber, acid detergent fiber, and acid detergent lignin) analyses were performed by the Ankom technology filter bag technique (Macedon, NY, USA), AOAC approved procedure Ba 6a 05. The mineral level by weight difference was assessed by placing samples into a high temperature muffle furnace where the temperature was maintained at 550°C. Carbohydrate percentage was calculated as complement to 100 of the sum of fat, protein, and minerals.

Total lipids (TLs) were extracted from 5 g of sample with a chloroform/methanol mixture (2:1, v/v) according to Rodriguez-Estrada et al. (6). TLs were separated into PLs, triglycerides, and free fatty acids (FFA) according to Kaluzny et al. (7), by means of SPE-NH_2_ cartridges (Supelclean 3 mL, Supelco Sigma-Aldrich, USA). Approximately 30 mg of TL was used. The sample was eluted with three subsequent eluents: chloroform/2-propanol (2:1, v/v), which led to neutral lipid (NL) separation, 2% acetic acid in diethyl ether, which separated FFA and methanol, from which PLs were obtained. NL, FFA, and PL were dried under N_2_ and reconstituted into 1 mL of hexane. TLs, NLs, and PLs were trans-esterified using a methanolic solution 0.5 N of sodium methoxide after adding the internal standard (C19:0 methyl ester). FA methyl esters were separated and identified by means of a GC-FID apparatus (GC 2000 plus, Shimadzu, Kyoto, Japan) equipped with a high polar capillary column (SP-2650, Sigma-Aldrich, St. Louis, MO, USA) according to Serra et al. (8).

The cholesterol was quantified according to Boselli et al. (9), with minor modifications. Briefly, an aliquot (250 mg) of TLs was dissolved in *n*-hexane:isopropanol (4:1, v/v) and saponified according to Hulshof et al. (10). Dehydrocholesterol (Steraloids, Newport, RI, USA) was used as the internal standard. The unsaponifiable matter was silylated by a solution of pyridine, hexamethyldisilazane, and trimethylchlorosilane (5:2:1, v/v/v). The silylated derivatives were injected into a GC-FID apparatus (GC 2000 plus, Shimadzu, Kyoto, Japan) equipped with a VF 1-ms apolar capillary column (RXI 1-ms, 30 m × 0.25 mm i.d., 0.25 µm film thickness; Restek, Bellefonte, PA, USA) according to Serra et al. (11).

### Statistical Analysis

Data were analyzed using the following linear model by JMP9 software (SAS Institute Inc., Cary, NC, 2010):

$$y_{ij}=\mu +{\text{muscle}}_{i}+{\text{breed}}_{j}+{\text{muscle}}_{i}\times {\text{breed}}_{j}+\varepsilon_{ij},$$

where *y_ij_* = variables (fatty acids, cholesterol, count, perimeter, and area of fiber); μ = media; muscle*_i_* = fixed effect of the *i*^th^ muscle (SM, TB); breed*_j_* = fixed effect of the *j*^th^ breed (Lm, Ma); ε*_ij_* = error.

Least-square means with their SEs were recorded, and treatment effects were declared significant at *P* < 0.05. Linear contrasts were tested by the *t*-test with Tukey’s adjustment. Relationship between lipid fractions was processed by the Pearson correlation coefficient to measure the degree of linear relationship between the variables.

## Results

Animals were approximately 19 months old at the time of slaughter (Ma 18.9 ± 0.9 months; Lm 18.5 ± 1.0 months). Live weight at the time of slaughter was significantly higher in Ma than in Lm (570.8 ± 15.3 vs 485.1 ± 17.2 kg, *P* < 0.01 for Ma and Lm, respectively); Ma also showed a higher daily weight gain with respect to Lm (1.10 ± 0.4 vs 0.92 ± 0.05 kg/day; *P* = 0.03).

Breed was also a significant variation factor for proximate composition. In fact, Ma muscle was higher in dry matter and lipid content than the Lm (Table 2). Due to the higher TL content, Ma was higher in NLs and lower in polar lipids than Lm (Table 2).

**Table 2:** Proximate composition and lipid fraction composition of SM and TB muscles of Ma and Lm breeds.

	Breed		Muscle		SE	Significance		
	Lm	Ma	TB	SM		Breed	Muscle	Breed × muscle
**Proximate composition**								
Dry matter (g/100 g of muscle)	24.3	26.4	25.2	25.4	0.6	*	ns	ns
TLs (g/100 g of mDM)	4.3	9.4	7.7	6.4	1.6	*	*	ns
Crude proteins (g/100 g of mDM)	86.0	85.1	87.3	83.8	0.7	ns	**	ns
Ashes (g/100 g of mDM)	4.7	4.1	4.4	4.4	0.2	*	ns	ns
Carbohydrates (g/100 g of mDM)	4.8	1.3	0.8	5.3	0.3	*	**	ns

**Lipid fractions**								
NL (g/100 g TL)	65.6	72.6	70.1	68.1	2.5	*	ns	ns
PL (g/100 g TL)	28.3	21.5	23.9	25.9	2.7	*	ns	ns
FFA (g/100 g TL)	1.6	1.9	2.1	1.5	0.4	ns	ns	ns

**Cholesterol content**								
Chol (mg/100 g muscle)	37.7	39.8	39.3	38.2	3.0	ns	ns	ns
Chol (g/100 g TL)	3.0	2.1	2.6	2.5	0.4	*	ns	ns

*Lm, Limousine; Ma, Maremmana; SM, Semimembranosus; TB, Triceps brachii; mDM, muscle dry matter; TL, total lipid; NL, neutral lipid; PL, phospholipid; FFA, free fatty acids; Chol, cholesterol*.

(*) 0.01 ≤ *P* < 0.05; (**) *P* < 0.01; ns, not significant.

Muscle significantly affected the carbohydrate and lipid contents, as the former was higher in SM than in TB, and the latter was higher in TB than in SM (Table 2).

Breed was also a significant variation factor for the histological characteristics (Table 3). The Lm muscle showed more and, consequently, smaller fibers than Ma. Thus, MSP was significantly lower in Lm than in Ma. Consequently, considering the total transversal muscle section, the Lm muscle showed a higher TSP than the Ma (Table 3).

**Table 3:** Muscle fibers histological characteristics of SM and TB muscles from Ma and Lm breeds.

	Breed		Muscle		SE	Significance		
	Lm	Ma	TB	SM		Breed	Muscle	Breed × muscle
TNF (No)	80.5	60.8	75.0	66.2	2.6	***	*	ns
CSA (μm^2^)	2,356	5,472	3,332	4,496	1,457	*	ns	ns
MSP (μm)	171.5	259.7	227.8	203.5	53.6	*	ns	ns
TSP (mm)	14.2	12.2	14.0	12.3	0.4	*	ns	ns

*Lm, Limousine; Ma, Maremmana; SM, Semimembranosus; TB, Triceps brachii; TNF, total number of muscle fibers; CSA, cross-sectional area; MSP, mean sarcolemma perimeter; TSP, total sarcolemma perimeter*.

(*) 0.01 ≤ *P* < 0.05; (***) *P* < 0.001; ns, not significant.

Table 4 shows the relationship between histological parameters and lipid composition. TL showed a strong negative correlation with PL, and a strong positive correlation with NL. On the other hand, TSP correlated negatively with TL and NL and correlated positively with PL. Finally, cholesterol correlated positively with TL and NL.

**Table 4:** Pearson correlations (above diagonal) and correlation significance (below diagonal).

	TSP	TL	PL	NL	Chol-m	Chol-TL
TSP	1.000	−0.505	0.658	−0.657	−0.383	0.720
TL	**	1.000	−0.900	0.808	0.828	−0.812
PL	*	***	1.000	−0.911	−0.697	0.940
NL	*	**	***	1.000	0.668	−0.844
Chol-m	ns	**	ns	ns	1.000	−0.530
Chol-TL	**	**	***	***	ns	1.000

*TSP, total sarcolemma perimeter; TL, total lipid; PL, phospholipid; NL, neutral lipid, Chol-m, cholesterol (mg/100 g of muscle); Chol-TL, cholesterol (g/100 g of TL)*.

(*) 0.05 < *P* ≤ 0.01; (**) 0.01 < *P* ≤ 0.001; (***) *P* < 0.001.

Phospholipids and NLs showed a specific fatty acid composition. PLs were high in PUFA, mainly represented by linoleic acid (C18:2 n-6), and in PUFA with acyl chain constituted by 20 carbon atoms or more. This fatty acid class consisted of about 20% of total fat and were mostly represented by arachidonic acid (C20:4 n-6), eicosapentaenoic acid (EPA, C20:5 n-3), docosapentaenoic acid (DPA, C22:5 n-3), and docosahexaenoic acid (DHA, C22:6 n-3) (Table 5). Triglycerides mainly contain monounsaturated fatty acids (MUFA), particularly oleic acid (C18:1 c9), and SFA, particularly palmitic acid (C16:0) while PUFA were poorly represented (Table 6).

**Table 5:** Fatty acid composition of PLs of SM and TB muscles from Ma and Lm breeds (g/100 g of FA from PL).

	Breed		Muscle		SE	Significance		
	Lm	Ma	TB	SM		Breed	Muscle	Breed × muscle
TL (g/100 g of muscle)	4.34	9.43	7.67	6.38	0.52	*	*	ns
PL (g/100 g of TL)	28.34	21.54	23.95	25.93	2.68	*	ns	ns
Total FA (g/100 g of FA from PL)	19.62	14.73	16.56	17.80	0.37	*	ns	ns

**Fatty acids**								
C15:0	0.15	0.51	0.24	0.41	0.14	ns	ns	ns
C16:0	21.74	21.68	22.14	21.28	1.38	ns	ns	ns
C17:0 -*iso*	0.45	0.35	0.55	0.25	0.07	*	ns	ns
C16:1 *c*7	0.57	0.48	0.34	0.71	0.13	ns	ns	ns
C16:1 *c*9	0.78	1.04	0.90	0.92	0.11	ns	ns	ns
C17:0	0.68	0.46	0.61	0.54	0.12	ns	ns	ns
C18:0 -*iso*	0.09	0.13	0.14	0.09	0.10	ns	ns	ns
C17:1 *c*9	0.52	0.50	0.49	0.53	0.12	ns	ns	ns
C18:0	7.03	7.11	7.10	7.03	0.46	ns	ns	ns
C18:1 *c*9	18.78	25.34	21.24	22.88	1.81	*	ns	ns
C18:1 *c*11	1.22	1.59	1.20	1.61	0.68	ns	ns	ns
C18:1 *c*12	0.57	0.47	0.53	0.51	0.12	ns	ns	ns
C18:2 n-6	24.64	20.37	23.62	21.39	1.37	*	ns	ns
C18:3 n-3	1.98	1.47	1.78	1.66	0.20	ns	ns	ns
C20:2 n-6	0.25	0.32	0.26	0.31	0.04	ns	ns	ns
C22:0	3.01	1.97	2.74	2.23	0.04	ns	ns	ns
C20:3 n-6	10.22	8.61	9.82	9.01	0.26	*	ns	ns
C20:4 n-6	0.13	0.07	0.14	0.06	0.75	ns	ns	ns
C23:0	1.83	2.84	1.46	3.21	0.04	ns	ns	ns
C20:5 n-3	0.87	1.42	0.86	1.44	1.85	ns	ns	ns
C22:4 n-6	3.23	2.45	2.92	2.76	0.60	ns	ns	ns
C22:5 n-3	0.43	0.36	0.31	0.49	0.29	ns	ns	ns
C22:6 n-3	0.15	0.51	0.24	0.41	0.20	ns	ns	ns

**Fatty acid classes**								
SFA	30.67	30.76	31.31	30.11	1.64	ns	ns	ns
MUFA	22.92	29.51	24.97	27.46	1.70	*	ns	ns
PUFA	46.42	39.73	43.72	42.44	2.66	ns	ns	ns

*C14:0, C14:1 c9, C16:0 -iso, C17:0 -ante, C18:1 t6–8, C18:1 t9, C18:1 t10, C18:1 t11, C18:1 t12, and C18:2 c9,t11 were detected but not included in the table because less than 0.2 g/100 g of FA*.

*Lm, Limousine; Ma, Maremmana; SM, Semimembranosus; TB, Triceps brachii; SFA, saturated fatty acids; MUFA, monounsaturated fatty acids; PUFA, polyunsaturated fatty acids; PL, phospholipid; TL, total lipid*.

(*) 0.01 ≤ *P* < 0.05; ns, not significant.

**Table 6:** Fatty acid composition of NLs of SM and TB muscles from Ma and Lm breeds (g/100 g FA from NL).

	Breed		Muscle		SE	Significance		
	Lm	Ma	TB	SM		Breed	Muscle	Breed × muscle
TL (g/100 g of muscle)	4.34	9.43	7.67	6.38	0.52	*	*	ns
NL (g/100 g of TL)	65.65	72.59	70.12	68.12	2.52	*	ns	ns
Total FA (g/100 g FA from NL)	62.04	68.92	66.80	64.16	0.18	*	ns	ns

**Fatty acids**								
C12:0	0.18	0.18	0.18	0.18	0.03	ns	ns	ns
C14:0	1.45	2.47	2.14	1.79	0.36	ns	ns	ns
C14:1 *c*9	0.56	0.86	0.71	0.71	0.11	ns	ns	ns
C15:0	0.31	0.30	0.30	0.31	0.03	ns	ns	ns
C16:0	22.63	24.11	23.21	23.54	1.23	ns	ns	ns
C17:0 -*iso*	0.30	0.27	0.29	0.28	0.02	ns	ns	ns
C16:1 *c*7	0.19	0.18	0.18	0.18	0.02	ns	ns	ns
C16:1 *c*9	4.84	5.30	5.10	5.04	0.38	ns	ns	ns
C17:0 -*ante*	0.13	0.19	0.15	0.17	0.02	*	ns	ns
C17:0	0.84	0.69	0.79	0.75	0.03	*	ns	ns
C17:1 *c*9	0.85	0.69	0.74	0.79	0.12	ns	ns	ns
C18:0	10.66	9.07	9.97	9.76	0.69	ns	ns	ns
C18:1 *t*6–8	0.16	0.14	0.16	0.14	0.02	ns	ns	ns
C18:1 *t*9	0.23	0.23	0.24	0.22	0.02	ns	ns	ns
C18:1 *t*10	0.53	0.34	0.46	0.41	0.08	ns	ns	ns
C18:1 *t*11	0.68	0.58	0.67	0.60	0.09	ns	ns	ns
C18:1 *t*12	0.25	0.22	0.23	0.24	0.03	ns	ns	ns
C18:1 *c*9	47.32	46.80	46.88	47.23	1.42	ns	ns	ns
C18:1 *t*15	1.97	2.13	2.09	2.02	0.17	ns	ns	ns
C18:1 *c*11	0.30	0.34	0.33	0.31	0.19	ns	ns	ns
C18:1 *c*12	0.38	0.48	0.43	0.43	0.03	ns	ns	ns
C18:1 *c*13	0.16	0.16	0.16	0.16	0.04	ns	ns	ns
C18:2 *t*9,*t*12	2.30	1.94	2.20	2.04	0.03	ns	ns	ns
C18:2 n-6	0.22	0.30	0.27	0.24	0.16	ns	ns	ns
C20:1 *c*11	0.33	0.34	0.34	0.33	0.04	ns	ns	ns
C18:3 n-3	0.29	0.30	0.30	0.29	0.03	ns	ns	ns
C18:2 *c*9,*t*11	0.22	0.13	0.22	0.14	0.04	ns	ns	ns
C23:0	0.18	0.18	0.18	0.18	0.03	ns	ns	*

**Fatty acid classes**								
SFA	37.37	38.02	37.86	37.53	1.87	ns	ns	ns
MUFA	59.27	59.01	58.93	59.35	1.69	ns	ns	ns
PUFA	3.47	3.09	3.33	3.24	0.26	ns	ns	ns

*C10:0, C14:0 -iso, C15:0 -iso, C15:0 -ante, C16:0 -iso, C17:0 -ante, C18:0 -iso, C18:2 t9,c13, C20:1 c8, C20:2 n-6, C22:0, C20:3 n-6, C20:4 n-6, C20:5 n-3, C22:4 n-6, and C22:5 n-3 were also detected but not included in the table because less than 0.2 g/100 g of FA from NL*.

*Lm, Limousine; Ma, Maremmana; SM, Semimembranosus; TB, Triceps brachii; SFA, saturated fatty acids; MUFA, monounsaturated fatty acids; PUFA, polyunsaturated fatty acids; TL, total lipid; NL, neutral lipid*.

(*) 0.01 ≤ *P* < 0.05; ns, not significant.

Breed was a significant variation factor with respect to total fatty acid composition (Table 7). Lm had a PUFA content that was almost twice that of Ma. This was mainly due to linoleic acid and to PUFA with a chain of 20 carbon atoms or more, both n-3 fatty acid series (DHA, DPA, and EPA) and n-6 series (arachidonic acid). The simultaneous increase in n-6 and n-3 fatty acid series did not change the n-6/n-3 ratio (Table 7). Ma, on the other hand, showed a high content of MUFA, particularly, of oleic acid (Table 7).

**Table 7:** Fatty acid composition of TL of SM and TB muscles from Ma and Lm breeds (g/100 g of TL).

	Breed		Muscle		SE	Significance		
	Lm	Ma	TB	SM		Breed	Muscle	Breed × muscle
TL (g/100 g of muscle)	4.34	9.43	7.67	6.38	0.52	*	*	ns

**Fatty acids**								
C14:0	0.82	1.67	1.23	1.27	0.25	ns	ns	ns
C14:1 *c*9	0.22	0.50	0.35	0.37	0.08	ns	ns	ns
C15:0	0.18	0.21	0.21	0.19	0.02	ns	ns	ns
C16:0	12.51	16.90	14.69	14.72	1.46	ns	ns	ns
C17:0 -*iso*	0.24	0.21	0.23	0.23	0.02	ns	ns	ns
C16:1 *c*9	2.03	3.05	2.52	2.57	0.32	ns	ns	ns
C17:0	0.46	0.51	0.49	0.47	0.04	ns	ns	ns
C17:1 *c*9	0.41	0.47	0.45	0.43	0.02	ns	ns	ns
C18:0	6.98	7.41	7.42	6.97	0.45	ns	ns	ns
C18:1 *t*10	0.26	0.20	0.25	0.21	0.03	ns	ns	ns
C18:1 *t*11	0.34	0.37	0.39	0.32	0.04	ns	ns	ns
C18:1 *t*12	0.17	0.14	0.17	0.14	0.01	ns	ns	ns
C18:1 *c*9	19.99	27.54	23.87	23.67	1.88	*	ns	ns
C18:1 *c*11	1.13	1.27	1.27	1.13	0.06	ns	ns	ns
C18:1 *c*12	0.22	0.24	0.24	0.23	0.02	ns	ns	ns
C18:1 *c*13	0.16	0.26	0.21	0.21	0.03	*	ns	ns
C18:2 n*-6*	5.47	3.24	4.59	4.12	0.41	**	ns	ns
C18:3 n-3	0.49	0.36	0.43	0.41	0.03	ns	ns	ns
C20:3 n-6	0.62	0.27	0.46	0.44	0.06	**	ns	ns
C20:4 n-6	2.12	0.97	1.62	1.47	0.22	*	ns	ns
C20:5 n-3	0.34	0.15	0.23	0.26	0.06	ns	ns	ns
C22:4 n-6	0.22	0.13	0.18	0.17	0.02	*	ns	ns
C22:5 n-3	0.72	0.34	0.52	0.53	0.10	*	ns	ns

**Fatty acid classes and index**								
SFA	21.65	27.52	24.82	24.35	2.19	ns	ns	ns
MUFA	25.56	34.83	30.44	29.95	2.36	*	ns	ns
PUFA	10.37	5.80	8.41	7.75	0.88	*	ns	ns
PUFA n-3	1.65	0.90	1.25	1.28	0.19	*	ns	ns
PUFA n-6	8.48	4.65	6.90	6.25	0.79	*	ns	ns
n-6/n-3	5.14	5.17	5.52	4.88	0.39	ns	ns	ns

*C10:0, C12:0, C14:0 -iso, C15:0 -iso, C15:0 -ante, C16:0 -iso, C16:1c7, C17:0 -ante, C18:0 -iso, C18:1 t6–8, C18:1 t9, C18:1 t15, C18:1 t16, C18:2 t9,t12, C18:2 t9,c13, C20:1 c8, C20:1 c11, C18:2 c9,t11, C20:2 n-6, C22:0, C23:0, and C22:6 n-3 were detected but not included in the table because less than 0.2 g/100 g of TL*.

*Lm, Limousine; Ma, Maremmana; SM, Semimembranosus; TB, Triceps brachii; SFA, saturated fatty acids; MUFA, monounsaturated fatty acids; PUFA, polyunsaturated fatty acids; TL, total lipid*.

(*) 0.01 ≤ *P* < 0.05; ns, not significant.

Breed significantly affected the lipid cholesterol content with a higher level in Lm than Ma (Table 2). There was no effect either of breed or muscle with respect to muscle cholesterol content (Table 2).

## Discussion

The higher weight at slaughtering and average daily gain of Ma with respect to Lm are interesting results considering that the feeding system was the same and that Lm is more specialized for beef production than Ma. These findings seem to indicate that environment factors dramatically affect the animal performance and limit the potentiality of most selected breeds. Thus, the choice of a breed for a marginal area requires a careful consideration, making the autochthonous breeds more suitable for rearing in these areas.

Due to higher muscle TL content, Ma was higher in NLs and lower in PLs than Lm. The differences in proximate composition between muscles could be due to their specific energetic metabolism. The muscle fiber metabolism was not the focus of the present experiment; however, it is well known that the energy of SM stems mainly from glycolytic pathways, while most of the TB energy is produced during the respiratory chain (12, 13). Therefore, SM and TB are classified as glycolic and oxidative muscles, respectively. Carbohydrates (namely, glycogen) are the main fuel of the glycolytic muscle, whereas lipids are the main fuel for the oxidative muscle (2).

On the whole, the histological properties of muscle fibers are mainly related to the animals’ age and, in particular, younger animals show smaller muscle fibers than the older animals (14). In this experiment, animals were slaughtered at the same age, thus the histological differences could be attributed to the somatic precocity of the animals. Our results seem to indicate that, in the present trial, Ma was more precocious than Lm.

These findings are quite surprising, considering that the Lm breed has been genetically improved for beef production and it is generally considered a more precocious breed than Ma. This thus led us to hypothesize that the rearing system dramatically affected the phenotypic expression of the two breeds. The Ma breed has adapted perfectly to the climatic and pasture conditions, which, conversely, are not as easy for a non-native breed. In fact, poor summer (SU) rainfall limited the grass growth and led to low quality pasture in SU and AU, thus warranting feed supplementation. As mentioned in the Section “Materials and Methods,” we formulated a ration aimed at obtaining an average daily gain of 1.1 kg with a feeding regimen that met the organic animal production rules. Thus, the metabolizability (the ratio between the crude and metabolizable energy) of the ration was low. Thus, it could be that the Ma breed fully exploited these rearing conditions, whereas the Lm reduced its growth rate.

The two breeds had different in TNF and CSA results, with a consequent variation in TSP. Sarcolemma has many major functions including signal transduction, membrane trafficking, and protein disposal. It contains several important lipid substances organized in a lipid raft phase [lipid ordered phase (Lo) of membrane], “immersed” in a non-raft matrix, which is a gel phase named the lipid-disordered phase—Ld (15). Animal selection for increased growth rate and lean meat content shifts the muscle metabolism toward a more white glycolytic and less red-oxidative type (16).

Lipid moiety is fundamental for protein deployment, receptor activation and for triggering the signaling flux, and thus is also important for muscle contraction. Sarcolemma also comprises PLs, which contain two molecules of unsaturated long chain fatty acids, linked to a glycerol backbone by an ester bond (O-acyl bond). Thus, both the TL deposition and histological characteristics of two breeds could explain the different percentage distribution of the lipid fractions. It thus seems that the environment conditions affected not only the animal performance but also their meat quality due to the fact that the different distributions of lipid fractions, in turn, affect the total fatty acid composition. In fact, PL and NL show a specific and very different fatty acid composition. PLs are directly involved in the cell metabolism and thus are high in PUFA, which have important functions in muscle fibers. For instance, in relation to membrane functionality, the fatty acid composition of sarcolemma could affect its insulin sensitivity (17). It has also been demonstrated that some PUFA may modulate the lipid microenvironment of certain enzymes (18). It is also well known that certain PUFA are substrates to produce eicosanoids, which regulate the inflammatory cascade (19), of endocannabinoids, which affect energy intake (20), and are also involved in the regulation of some cardiovascular heart diseases (CHD) (21). On the other hand, in the organism and in muscle cells, NLs play an energy storage role, and thus provide above all monounsaturated and SFA. Oleic acid, the main MUFA, has many positive health effects for human, while SFA are generally detrimental for human health (22).

The effects on human health of these fatty acids cannot be considered separately from those of cholesterol. Cholesterol is a very important substance for the animal organism, for which there is a very effective homeostasis system (active absorption, transcriptional and posttranscription control of the synthesis, lipoprotein blood transport, membrane receptors for the transport of lipoproteins, recycling of intestinal bile salts). With respect to the cell membrane, cholesterol is a functional necessity for the formation of microdomains, which perform very important cell functions.

However, due to alterations to the homeostasis system, cholesterol can accumulate in blood vessels and promote CHD (23). In these conditions, cholesterol from food could negatively affect serum cholesterol levels and thus homeostasis health. Thus, in the past few years, consumers have become increasingly concerned about the possible negative health effects of food from animals and particularly from red meat. It is therefore important to define the mechanism determining the cholesterol content in the muscle.

The lack of significance of muscle cholesterol content is a surprisingly result. In fact, as cholesterol is mainly contained in membrane cells, and due to the higher TSP in Lm, it should have been higher in Lm than in Ma. It is possible that the high TL content in the Ma breed masked the correlation between the cholesterol and sarcolemma total perimeter. On the other hand, when the cholesterol content is expressed on a TL basis, after the elimination of the effect of the TL content, the breed represents a significant variation factor (Lm showed a higher cholesterol content). In our experiment cholesterol showed a strong positive correlation with TSP (and with PL), only when it was expressed as g/100 g of TL. This led us to speculate that the muscle cholesterol content is related to TSP, but that TL and NL may hide this effect.

In conclusion, this study highlights the importance of the environment, climatic and rearing conditions in term of how they affect the full expression of the genotypic potential of a bovine breed both in relation to animal performance and meat quality. This evidence should be considered in the introduction of a bovine breed in a new area, and in the development of genetic improvement programs aimed at producing animals that are suitable for rearing in marginal areas where resources are poor.

## Ethics Statement

All the experimental procedures used in this study, followed the EU guidelines for the care and use of animals in research (Italian official bulletin no. 61, 2014).

## Author Contributions

AS, GC, EG, and MM: conceived and designed the experiments. LC, CL, AB, FC, and AC: performed the experiments.

## Conflict of Interest Statement

The authors declare that the research was conducted in the absence of any commercial or financial relationships that could be construed as a potential conflict of interest.
